# Clinical Performance of an Ultrahigh Resolution Chromosomal Microarray Optimized for Neurodevelopmental Disorders

**DOI:** 10.1155/2016/3284534

**Published:** 2016-11-16

**Authors:** Karen S. Ho, Hope Twede, Rena Vanzo, Erin Harward, Charles H. Hensel, Megan M. Martin, Stephanie Page, Andreas Peiffer, Patricia Mowery-Rushton, Moises Serrano, E. Robert Wassman

**Affiliations:** ^1^Lineagen, Inc., Salt Lake City, UT, USA; ^2^Department of Pediatrics, University of Utah, Salt Lake City, UT, USA

## Abstract

Copy number variants (CNVs) as detected by chromosomal microarray analysis (CMA) significantly contribute to the etiology of neurodevelopmental disorders, such as developmental delay (DD), intellectual disability (ID), and autism spectrum disorder (ASD). This study summarizes the results of 3.5 years of CMA testing by a CLIA-certified clinical testing laboratory 5487 patients with neurodevelopmental conditions were clinically evaluated for rare copy number variants using a 2.8-million probe custom CMA optimized for the detection of CNVs associated with neurodevelopmental disorders. We report an overall detection rate of 29.4% in our neurodevelopmental cohort, which rises to nearly 33% when cases with DD/ID and/or MCA only are considered. The detection rate for the ASD cohort is also significant, at 25%. Additionally, we find that detection rate and pathogenic yield of CMA vary significantly depending on the primary indications for testing, the age of the individuals tested, and the specialty of the ordering doctor. We also report a significant difference between the detection rate on the ultrahigh resolution optimized array in comparison to the array from which it originated. This increase in detection can significantly contribute to the efficient and effective medical management of neurodevelopmental conditions in the clinic.

## 1. Introduction

Neurodevelopmental disabilities, including developmental delay (DD), intellectual disability (ID), and autism spectrum disorder (ASD), affect up to 15% of children [1]. However, in the majority of cases, a child's clinical presentation does not allow for a definitive etiological diagnosis. Copy number variants (CNVs) contribute significantly to the etiology of neurodevelopmental disorders, as well as syndromes of multiple congenital anomalies (MCA). The clinical utility of chromosomal microarray analysis (CMA) for the detection of CNVs associated with these disorders has been recognized by multiple professional societies and has been deemed the first-tier clinical diagnostic test for the evaluation of these disorders [2–6].

Microarrays of various designs and reflective of variable genomic content have been applied to the clinical care of individuals with these conditions; as such, there are varying degrees of diagnostic yield with an increase over time as arrays have evolved [7–17]. The ACMG issued a guideline in 2011 on the optimal design of CMAs and recommended inclusion of additional probe content in areas of known relevance [18]. Most studies reporting on the clinical performance of CMA have been on populations enriched by virtue of the nature of the reporting institution and relative indications for testing.

This study summarizes the results of routine clinical CMA testing in a CLIA-certified laboratory using an array specifically designed to increase detection of CNVs in genomic regions of demonstrated relevance to DD/ID/ASD over a period of 3.5 years.

## 2. Materials and Methods

### 2.1. Patient Ascertainment

Data were obtained from a consecutive series of routine clinical samples referred for CMA to a CLIA-licensed laboratory for etiological diagnosis of DD/ID/ASD and MCAs between July 2012 and December 2015. Patients selectively ascertained and tested as a part of research studies were excluded from these analyses to preclude bias in the observed rates of diagnosis. A second smaller series of 1194 CMAs performed on the same cohort (i.e., identical referral base and underlying patient demographics) with the Affymetrix CytoScan® HD array run during development and local regulatory approval periods is compared here as well to control for the likely ascertainment bias present in previously published reports. Testing indications used here to group patients are defined by the codes routinely provided by referring physicians when ordering tests and are derived from the International Classification of Diseases, Clinical Modification, Revisions 9 or 10, (ICD-9 and ICD-10) from the Centers for Medicare & Medicaid Services (https://www.cms.gov/).

### 2.2. Microarray Design

The custom microarray [FirstStepDX PLUS® (FSDX PLUS®), Lineagen, Inc.] was utilized in this study in all cases except where specified, and its analytical and clinical validation has been described in detail elsewhere [19]. It is an expanded whole genome chromosomal microarray (CMA) built upon the ultrahigh resolution Affymetrix CytoScan HD platform plus 88,435 custom probes targeting genomic regions strongly associated with ID/DD/ASD [15–24] added under good manufacturing practices (GMP) by Affymetrix using their previously described microarray design process [16]. This resulted in a grand total of 2,784,985 probes. Both copy number (CNV) and single nucleotide polymorphic (SNP) probes are included in the array, which is consistent with the ACMG guideline for CMA design, as is the “enrichment of probes targeting dosage-sensitive genes known to result in phenotypes consistent with common indications for a genomic screen” [18]. Such critical regions that did not contain ≥1 probe/1000 bp on the baseline array were supplemented with additional probe content to provide improved detection of smaller deletions and duplications. Additional probe enrichment targeted genomic regions identified by our prior studies and identified elsewhere in the medical literature. These regions included published copy number variants and individual genes associated with DD/ID/ASD [20–29]. The increase in analytical sensitivity resulting from this additional 3.3% probe content has been calculated to be 2.6% [19].

### 2.3. CMA Performance and Interpretation

CMA was routinely performed on DNA extracted by standard methodologies from buccal swab samples (ORAcollect®) in a CLIA-certified laboratory. CMA reagents and equipment were as specified by Affymetrix. The established standard cytogenetic criteria for interpretation were routinely applied [30] with minimum of 25-consecutive impacted probes as the baseline determinant for deletions and 50 probes for duplications. Rare CNVs (<1% overall population frequency) were determined to be “pathogenic” if there was sufficient published clinical evidence (at least two independent publications) to indicate that haploinsufficiency or triplosensitivity of the region or gene(s) involved is causative of clinical features. If, however, such clinical evidence was insufficient, but at least some preliminary evidence existed for a causative role for the region or gene(s) therein, and they were not previously categorized as normal population variants in the Database of Genomic Variants (DGV) [31], they were classified as variants of unknown significance (VOUS). Areas of absence of heterozygosity (AOH) were also classified as VOUS if they were of sufficient size and location to increase the risk for conditions with autosomal recessive inheritance or conditions with parent-of-origin/imprinting effects. Cases with no CNVs or only CNVs determined by these criteria to most likely represent normal population variants, for example, contained in databases such as DGV documenting presumptively benign CNVs, were reported as normal.

## 3. Results

### 3.1. Overall Findings and Diagnostic Yield

A total of 5487 FSDX PLUS CMAs were performed in this time period. There were 1558 females and 3929 males (M : F: 2.5 : 1) tested with a mean age of 7.2 years (median 5.5 years) (Table 1). While largely targeting a pediatric population, a subset of 225 patients was comprised of adults over 18 years old (parental and sibling studies excluded). Based on ICD-9 and ICD-10 codes at the time of referral, 3134 cases represented patients with intellectual (ID) or developmental (DD) disability of varying degrees, 3016 cases represented patients with ASD with or without other features, 743 cases represented patients with multiple congenital anomalies, and 1507 cases represented patients with speech/language delay. Referring physicians were pediatricians (15.0%), medical geneticists (11.2%), pediatric neurologists (40.2%), developmental pediatricians (31.6%), psychiatrists (1.7%), and other medical practitioners (0.4%).

**Figure 1:**
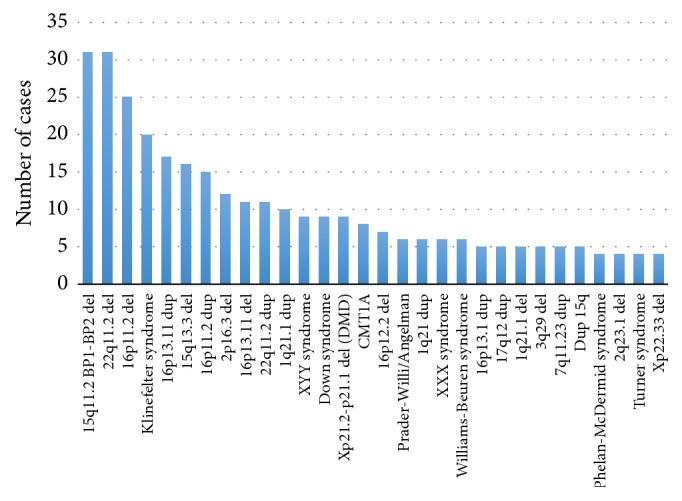
Most common pathogenic findings on 5487 chromosomal microarrays (FSDX PLUS).

The most common pathogenic findings detected in this unselected population of individuals with neurodevelopmental disorders are shown in Figure 1.

Overall, there were 506 (9.2%) pathogenic abnormalities and 1109 (20.2%) VOUS observed or a 29.4% overall CNV diagnostic yield for potentially abnormal findings (Table 1). However, the yield of pathogenic findings varies significantly on a multivariate basis including but not limited to referring physician specialty, age of patient at testing, patient gender, and referring indication or combination of indications. In addition, a single individual with a reported CNV may have more than one pathogenic CNV, a pathogenic CNV as well as a VOUS, or multiple VOUS findings in the same patient. Patients with any reportable finding had on average 1.2 CNVs per report. Of these, there were 13.4 CNVs classified as pathogenic and 23.2 CNVs classified as a VOUS per 100 CMAs (Table 2).

Rates vary significantly by the specialty of the ordering physician (Table 3), but, regardless of specialty expertise, clinically significant rates of detection were observed in all specialties as well as in the primary care setting. At the extremes were psychiatrists (5.5% diagnostic yield) and medical geneticists (15.5% diagnostic yield), and these groups also differed significantly in the rate of VOUSs (30% and 20%, resp.).

Reported duplications are significantly larger than deletions on average (Table 4). For both duplications and deletions, the average size of pathogenic CNVs was significantly larger than CNVs classified as a VOUS (*p* < 0.0001, two-tailed unpaired *t*-test).

### 3.2. Detection Rates by Indication and Age

In patients where the indication for testing was either DD/ID or MCA, the rate of pathogenic CNVs was highest in the first year of life at 16.8% and 21.3%, respectively (Tables 5 and 6). Values were lower but consistent throughout the remainder of childhood but peaked again in the small subset of adult patients tested at levels similar to the first year of life (16.8% and 20.0%, resp.).

Due to the age of clinical recognition, indications including ASD and speech/language deficits were not stratified as to the first year of life separately, but rather with a 0–3.4-year range as the lowest cohort considered. Patients with indications of speech/language deficits demonstrated a gradual rise in the rate of pathogenic findings from the 0–3.4-year-old group (6.7%) to peak in later childhood (12.8%), then dropping slightly in adolescence (10.8%) and reaching a maximum in the adults tested (19.1%) (Tables 7 and 8). VOUS rates were the highest in the youngest cohort (22.2%) and relatively constant in the other age groups but distinctly the lowest in the adults (14.9%).

Individuals with ASD as an indication for testing had a lower pathogenic yield but comparable VOUS rates to other categories (Table 7). The pathogenic rate rose gradually from 3.8% in the youngest cohort (0–3.4 years) to a peak at 8.7% in adolescence. The overall reported CNV rate for individuals with ASD ranged within 22%–29%, again with the peak in adolescents tested. Those with ASD not only had lower, albeit substantial, pathogenic CNV rates than those with other indications but also clearly lowered the rate for all other indications when it was an additional indication; for example, DD/ID/MCA rate when ASD ICD-9/ICD-10 code was excluded was 13.4% (Table 9). The diagnostic yield excluding ASD is significantly higher (*p* < 0.0001) than for the ASD cohort (13.4% compared to 5.9%, resp.).

VOUS rates tended to be relatively constant across groups and with age (18–22%) with the exception of a significantly lower rate in the first year of life for those with DD/ID indication (15.8%), which could be due to the small sample size (Table 5), and adults with MCAs or speech/language deficits (14.3% and 14.9%, resp.) (Tables 6 and 8). Those with MCAs also showed higher peak rates of 24.6% and 25% in the early childhood (1–3.4 years) and late childhood (5.5–10.1 years) cohorts and a dip, again potentially due to small sample size in this group, to 15.8% between these ages (Table 6).

### 3.3. Comparison to Detection on Baseline Array

Detection rates in the same overall cohort (i.e., same referral base, underlying patient demographics timeframe, laboratories, and interpretation process and criteria) on the CytoScan HD array (*N* = 1194), which was the baseline for FSDX PLUS, were lower than those in this series diagnosed on the custom FSDX PLUS array (9.0% pathogenic CNV and 14.2% VOUS compared to 9.2% and 20.2%, resp.) (Table 10).

## 4. Discussion

CMA is the guideline-recognized first-tier test in the evaluation of MCA, DD/ID, and ASD, [2–6] and yields significant rates of abnormal or potentially abnormal (VOUS) results [7–17] with clinical utility for the management of individuals with these disorders [28, 29, 32–39]. Since the introduction of this technology, the total genomic content in terms of probes on CMAs has progressively increased, leading to higher diagnostic yields and resolution of abnormalities [10, 14–17] with corresponding increases in clinical value of these tests [32–40]. In addition to guidelines on the clinical indications for CMA, ACMG has issued guidance on the appropriate content and design of such arrays and specifically opined that “it is desirable to have enrichment of probes targeting dosage-sensitive genes known to result in phenotypes consistent with common indications for a genomic screen (e.g., intellectual disability, developmental delays, autism, and congenital anomalies)” [18]. We report here on over three years' experience with a unselected clinical referral base on a CMA specifically designed to extend the scope of detection for individuals with ASD and other neurodevelopmental disorders through the addition of probes targeting genomic regions more recently identified as of pathogenic relevance to these disorders.

Our data demonstrate that diagnostic yield is a complex multivariate function dependent upon several clinical variables including the patient's clinical diagnosis/presentation, age at testing, and referring physician specialty training. An unselected consecutive referral base, with a substantial nonspecialty physician referral component, lack of bias toward selected subgroups (e.g., exclusion of research enriched population of WHS/4p-cohort in the present series) [41], and the active offering of testing to the most recent clinical indication for CMA, ASD, which has an expectably lower rate of such findings [13–15], would be expected to result in a lower overall diagnostic yield in the present series. However, the overall detection rate for clinically established pathogenic CNVs of 9.2% is equivalent or higher than other reported series/platforms [7–17] despite the inherent bias toward lower rates based on the unselected referral base and focus on ASD. An internal comparison to cases run on the standard array (CytoScan HD) which was the baseline for development of the FSDX PLUS array showed a slight, but not significant, increase in detection rate for pathogenic variants from 9.0% to 9.2% over the same referral base and underlying patient demographics, using the same interpretation paradigm. The same comparative analysis showed a highly significant differential in detection of VOUS from 14.2% to 20.2% (Chi-squared *p* value <0.0001). The analytical sensitivity of the FSDX PLUS array was recently calculated to be at least 2.6% greater than the baseline array, which is generally consistent with the observed increase in the overall rate of reportable CNVs (pathogenic plus VOUS) [19].

When individuals with ASD are excluded so as to more closely match populations reported for other CMA platforms/series, the diagnostic yield is further differentiated with diagnostic yields of 13.4% pathogenic and 19.4% VOUS and a total detection rate for potentially causative variants, of nearly 33%. It is likely that, even after this correction, other enrichment biases remain in comparing other series to this one.

While significantly lower than the overall population or the ASD-excluded subpopulation (*p* < 0.0001), the diagnostic yield in ASD cases of 5.9% pathogenic and 19.0% VOUS exceeds those previously reported [13–15] and supports the value of incremental targeted content for areas of clinical relevance in this important setting for CMA.

The variations in diagnostic yield evident in subgroup analyses may in turn contain clues for future research and causation. For example, the rise in rate of detected abnormalities in the ASD population with age suggests that earlier use of CMA and perhaps other genetic testing may be important. It is estimated that at least 20% of ASD individuals have an underlying genetic syndrome, but a survey of a large autism center showed that less than 10% of their population had received any form of genetic evaluation [42, 43].

Not surprisingly, patients who are tested in their first year of life for most “indication” groups have the highest diagnostic yield. This is likely due to the probability that increased severity of features would prompt physician investigation earlier in life. It is, however, remarkable that adults (>18 years old) tested also have such a high pathogenic CNV rate observed. This could be due to the relatively small size of this cohort. Alternatively, it may be more reflective of severity in that particular age group. For example, clinicians/families might believe that testing is not as valuable for adults but perform it anyway when the individual is considered to be relatively severely impaired.

In addition to clinically well-defined pathogenic CNVs, a variety of CNVs of less obvious correlation with causation are routinely found on all CMAs. Efforts to better identify and biologically define the relevance of VOUS in these disorders have critical importance to understanding disease mechanisms and, ultimately, give insight to appropriate medical management in the future. An increased rate of CNVs classified as VOUS is therefore of potential clinical importance. Furthermore, VOUS results have been clearly demonstrated to be of great importance to parents of patients with DD/ID/ASD [44–47].

While earlier literature did not typically consider VOUS in the diagnostic yield, this was due to inconsistent criteria for reporting, lack of established databases of normal population variants, and limited sharing of data [12, 13]. Today with these tools better established, it is common and reasonable to consider VOUS in an overall diagnostic yield [9, 32] as many of these variants will evolve into clearly pathogenic finding based on emerging clinical experience and represent an exciting and abundant opportunity to better understand the full range of genomic abnormalities contributing to the neurodevelopmental phenotypes.

Numerous studies have now demonstrated the clinical actionability and utility of CMA testing [32–40]. The increased yield of an optimized array as described here will extend the range and scope of this utility, and it is readily demonstrated through relevant case studies and series to date [35–40]. Of critical importance is the ongoing evaluation of novel methods to assess the potential role of VOUS findings in the underlying pathology of individual patients to realize the maximum benefit of the increased detection rate achieved through array and interpretation optimization.

## Figures and Tables

**Table 1:** Overall diagnostic yield of 5487 chromosomal microarrays in a routine clinical population.

	CMAs	Pathogenic (% yield)	VOUS (% yield)	Normal (% yield)
Total	5487	506 (9.2)	1109 (20.2)	3872 (70.6)
Female	1558	217 (13.5)	325 (20.2)	1065 (66.3)
Male	3929	342 (8.6)	797 (20.1)	2825 (71.3)

**Table 2:** Multiple CNV are observed in individual patients (mean 1.2 per patient).

	Total number of individual CNVs detected	CNVs per 100 tests (*N* = 5487)
ABN	734	13.4
VOUS	1272	23.2

**Table 3:** Diagnostic yield and mean patient age vary significantly by the specialty of the ordering physician.

Specialty	% total CMAs ordered	Average age (years) [6.4 overall]	Pathogenic % yield	VOUS % yield	Normal %
Pediatric neurology	40.2%	6.5	8.2%	20.0%	71.8%
Developmental and behavioral pediatrics	31.6%	6.1	7.1%	20.6%	72.3%
Pediatrics	15.0%	6.8	11.2%	17.6%	71.2%
Genetics	11.2%	6.0	15.5%	20.1%	64.4%
Psychiatry	1.7%	10.7	5.5%	29.7%	64.8%
Other specialties	0.4%	8.3	13.6%	18.2%	68.2%

**Table 4:** Clinically reported duplications are significantly larger than deletions on average.

		Deletions	Duplications
Pathogenic CNVs	Average size (kb)	3,284 (*N* = 474)	8,105 (*N* = 258)
	Median size (kb)	1,418	1,680

VOUS CNVs	Average size (kb)	308 (*N* = 584)	528 (*N* = 751)
	Median size (kb)	129	357

**Table 5:** Diagnostic yield by age in ID/DD (986 females and 2148 males, total *n* = 3134).

Age in years	Total tests	Pathogenic (% yield)	VOUS (% yield)	Normal (%)
0-1	95	16 (16.8%)	15 (15.8%)	64 (67.4%)
1–3.4	950	87 (9.2%)	188 (19.8%)	675 (71.1%)
3.5–5.4	572	54 (9.4%)	103 (18.0%)	415 (72.6%)
5.5–10.0	775	92 (11.9%)	152 (19.6%)	531 (68.5%)
10.1–18	623	65 (10.4%)	117 (18.8%)	441 (70.8%)
18+	119	20 (16.8%)	26 (21.8%)	73 (61.3%)
Total	3134	334 (10.7%)	601 (19.2%)	2199 (70.2%)

**Table 6:** Diagnostic yield by age in MCA (289 females and 454 males, total *n* = 743).

Age buckets	Total tests	Pathogenic (% yield)	VOUS (% yield)	Normal (%)
0-1 years	122	26 (21.3%)	23 (18.9%)	73 (59.8%)
1–3.4 years	179	29 (16.2%)	44 (24.6%)	106 (59.2%)
3.5–5.4 years	95	14 (14.7%)	15 (15.8%)	66 (69.5%)
5.5–10.4 years	164	30 (18.3%)	41 (25.0%)	93 (56.7%)
10.5–18	148	28 (18.9%)	29 (19.6%)	91 (61.5%)
18+	35	7 (20.0%)	5 (14.3%)	23 (65.7%)
Total	743	134 (18.0%)	157 (21.1%)	452 (60.8%)

**Table 7:** Diagnostic yield by age in ASD (622 females and 2394 males, total *n* = 3016).

Age in years	Number of tests	Pathogenic (% yield)	VOUS (% yield)	Normal (%)
0–3.4	735	28 (3.8%)	134 (18.2%)	573 (78.0%)
3.5–5.4	688	33 (4.8%)	121 (17.6%)	534 (77.6%)
5.5–10	789	50 (6.3%)	158 (20.0%)	581 (73.6%)
10.1–18	679	59 (8.7%)	138 (20.3%)	482 (71.0%)
18+	125	8 (6.4%)	25 (20%)	92 (73.6%)
Total	3016	178 (5.9%)	576 (19%)	2262 (75%)

**Table 8:** Diagnostic yield by age in speech/language deficits (427 females and 1080 males, total *n* = 1507).

Age buckets	Total tests	Pathogenic (% yield)	VOUS (% yield)	Normal (%)
0–3.4 years	449	30 (6.7%)	100 (22.2%)	319 (71.0%)
3.5–5.4 years	331	27 (8.2%)	63 (19.0%)	241 (72.8%)
5.5–10.4 years	420	52 (12.4%)	89 (21.2%)	279 (66.4%)
10.5–18	260	28 (10.8%)	50 (19.2%)	182 (70.0%)
18+	47	9 (19.1%)	7 (14.9%)	31 (66.0%)
Total	1507	146 (9.7%)	309 (20.5%)	1052 (69.8%)

**Table 9:** Diagnostic yield by age in neurodevelopmental disorders and/or MCA, excluding ASD (females = 909; males = 1486; total *n* = 2395).

Age in years	Total (excluding ASD)	Pathogenic (% yield)	VOUS (% yield)	Normal (%)
0–1	204	38 (18.6%)	37 (18.1%)	129 (63.2%)
1–3.4	699	84 (12.0%)	146 (20.9%)	469 (67.1%)
3.5–5.4	344	43 (12.5%)	63 (18.3%)	238 (69.2%)
5.5–10	589	82 (13.9%)	121 (20.5%)	386 (65.5%)
10.1–18	461	55 (11.9%)	83 (18.0%)	323 (70.1%)
18+	98	19 (19.4%)	15 (15.3%)	64 (65.3%)
Total	2395	321 (13.4%)	465 (19.4%)	1609 (67.2%)

**Table 10:** Comparison of FSDX (*N* = 5487) to CytoScan HD (*N* = 1194) arrays performed on same ascertainment base and interpretation paradigm.

Array	Pathogenic yield	VOUS yield	Normal
FSDX PLUS (*N* = 5487)	9.2%	20.2%	70.6%
CytoScan HD (*N* = 1172)	9.0%	14.2%	76.7%
